# A German View of the Qualifications of a Physician

**Published:** 1881-03

**Authors:** Albert S. Tadler


### A GERMAN VIEW OF THE QUALIFICATIONS OF A PHYSICIAN.

BY ALBERT S. TADLER, M. D.

Verily the vocation of the earnest physician has always been difficult, and it will be more difficult still if he devotes his time chiefly to social and humane pursuits. By no means does it suffice to study the structure of the human body and its functions in health and disease, to practice in surgical technology and the pharmaceutical art of prescribing. The physician must penetrate the thousand fold relations of civilized society, in its highest and lowest spheres. He must be conversant with all the intellectual processes and their condition of development, and must have a clear view of the results and limits of all natural science. To him belongs the "nihil humani a me alienum" in its fullest comprehension. He must possess not only the eye of an acute searcher, but all his mental powers must be educated in an efficient and harmonious manner, not less in the direction of sensibility than of intellect. This can not be effected by many years of university study. His physiological schooling must begin much earlier and must continue much later. On earth there is no object of study greater and more beautiful than man. He is the most difficult and the most sublime subject for thought and work. You must bring to your task a clear eye and sharp ears; acuteness of observation and patience for infinite study; an unclouded brain and an iron will strengthened in difficulty and embarrassment, but a warm, loving heart which takes cognizance of all sorrow and sympathizes with it; religious conviction and moral stamina which resist the seductions of sensuality, money and honors. Besides, you must have a respectable exterior; you must be polished in conversation; dexterous with the hands; possessed of health of body and soul. All this you must be if you would avoid misfortune and failure as a physician. You must carry the camel's burthen of knowledge and preserve the freshness of the poet. You must weigh all the tricks of charlatanism and in the mid-t of temptation remain an honest man. Remember that on your calling depends everything."

[After reading the above once, read it again, and then think about it. Almost every point there presented is as applicable to the dentist as to the general physician.

One of the great disabilities that attach to the dental profession is a want of appreciation of the qualities and attainments here referred to.—Ed. Reg.]

