# Correction

**Published:** 1881-06

**Authors:** 


### CORRECTION.

In the March number of the *Dental Register*, on page 126, is published a selection, entitled: "A German view of the qualifications of a Physician," for which the "Pacific Medical and Surgical Journal" should have had credit.

The article was translated from the German, by Albert S. Adler, M. D., instead of being written by Albert S. Tadler, M. D., as published in the Register. We beg everybody's pardon for permitting our proof-reader to make such a blunder, and trust the like will not occur again.

